# Prevalence and contributing factors of dental caries of 6-year-old children in four regions of China

**DOI:** 10.7717/peerj.6997

**Published:** 2019-05-31

**Authors:** Zhaoyou Wang, Wensheng Rong, Ying Zhang, Xiaojuan Zeng, Zhiqiang Li, Zhiwen Liu

**Affiliations:** 1 Department of Preventive Dentistry, Peking University School and Hospital of Stomatology, Beijing, China; 2 Department of Preventive Dentistry, Shanghai Stomatological Hospital, Fudan University, Shanghai, China; 3 Department of Preventive Dentistry, Stomatological Hospital of Guangxi Medical University, Nanning, Guangxi, China; 4 Department of Preventive Dentistry, School of Stomatology, Northwest Minzu University, Lanzhou, Gansu, China; 5 Department of Stomatology, The Second Xiangya Hospital of Central South University, Changsha, Hunan, China

**Keywords:** Dental caries, Epidemiology, Contributing factors, Children

## Abstract

**Background:**

From 2005 to 2015, the prevalence of dental caries in both primary and permanent dentitions was significantly increased in China. Previous studies have shown that the prevalence of permanent dental caries in school-aged children had already reached 19.7%–54.0%, 97.5% affecting first permanent molars. This study aimed to investigate the prevalence and contributing factors of dental caries in 6-year-old children in four regions of China to provide information for oral health promotion programs.

**Methods:**

A randomized cluster sampling method was employed in the study. All 6-year-old first grade children from the selected schools were invited to receive a clinical oral examination. Dental caries were diagnosed according to the World Health Organization criteria. The erupting first permanent molars were recorded using the modified International Caries Detection Assessment System. Questionnaires assessing children’s oral health-related behaviors and their caretakers’ oral health awareness and attitudes were completed by the children’s parents or guardians.

**Results:**

Overall, 4,936 6-year-old school children participated in the survey. The prevalence of caries among these children was 87.7%, with a mean number of decayed, missing and filled teeth of 6.04 (SD, 4.24). In primary dentition, the caries prevalence was 87.7%, and the mean dmft score was 6.01 (SD, 4.22). In permanent dentition, the caries prevalence was 2.0%, the mean DFS score was 0.04 (SD, 0.31). All permanent dental caries occurred on the first permanent molars. Carious tooth surfaces were identified as modified ICDAS code “A” to indicate initial caries and distinct visual change in enamel. The mean D_A_S score of non-cavitated caries in the first permanent molars was 0.18 (SD, 0.67). Logistic regression analysis showed that regional and gender factors were significantly related to the caries experience of these children.

**Conclusions:**

The 6-year-old children from four regions of China had sever primary dental caries and the first permanent molars were at high risk for dental caries. It is critical to protect permanent teeth from caries as early as the eruption of the first permanent molars.

## Introduction

From 2005 to 2015, the prevalence of dental caries in both primary and permanent dentitions significantly increased in China. The prevalence of caries in primary teeth of 5-year-olds increased from 66.0% to 71.9%, and the mean dmft score rose from 3.50 to 4.24 (Qi, 2008; Wang, 2018). The prevalence of caries in permanent teeth of 12-year-olds increased from 28.9% to 38.5%, and the mean DMFT score rose from 0.54 to 0.86 (Qi, 2008; Wang, 2018). From a global perspective, the prevalence of dental caries is different from country to country. The World Health Organization (WHO) uses the permanent caries experience in 12-year-olds as an important criterion to measure the severity of caries, which is divided into five levels (WHO, 1996). From 1980 to 2015, the global caries status showed a significant decline in the first 20 years and a slow upward trend in the more recent 15 years (Oral Health Database). In the fourth Chinese national oral health survey in 2015, the mean DMFT score of 12-year-olds was 0.86 (Wang, 2018), which was still in the very low level. However, from 2005 to 2015, each person had an average increase of 0.32 decayed teeth in China, with an alarming increase rate of 59.3%. In the mid-term and long-term plan for prevention and treatment of chronic diseases (2017–2025) issued by the General Office of the State Council of China, the goal for caries prevention is that the prevalence of caries in 12-year-old children should be under 30% by 2025 (General Office of the State Council of China, 2017), which seems to be a big challenge. Previous epidemiological surveys in various regions in China showed that the prevalence of permanent caries in school-aged children was between 19.7% and 54.0% (Cheng, 2009; Qing et al., 2011; Yan et al., 2010; Quanchun et al., 2012). Jiang et al. found the prevalence of caries in permanent teeth in children aged 7, 8 and 9 years was 16.9%, 19.0%, and 21.2%, respectively (Quanchun et al., 2012). In this study, many permanent teeth had already been cavitated by the age of 7, and the decay increased with age. Therefore, it is necessary to implement effective preventive measures before children reach the age of 7. 97.5% of caries in permanent teeth were found in the first permanent molars from the ages of 7–9 (Quanchun et al., 2012). In addition, since first permanent molars are critical for the establishment of occlusion, they should be the key for permanent caries prevention at the age of 6 and during the early stage of mixed dentition. The first permanent molars begin to erupt at the age of 6 (Kim Rud & Jette, 2010). It is important to understand the caries experience of 6-year-old children and the contributing factors. Therefore, this study analyzed the results of an oral examination and questionnaires of children sampled in four regions of China from October to December, 2014.

## Materials & Methods

### Sample selection

This study was carried out in Liaoning, Guangxi, Gansu and Hunan Province. A randomized cluster sampling method was used. First, in each of the four provinces, one county-level city was randomly selected. Second, half of the districts of each county-level city were randomly selected and all big-scale public primary schools (the number of students was ≥450) of the selected districts were included in the study. All 6-year-old first grade children of the selected schools were invited to participate in clinical oral examination in the selected schools. The study protocol was approved by the Ethics Committee of the Chinese Stomatological Association (approval number: 2014-003). Children in good general health with written informed consent form from their parents or guardians were selected.

### Caries examination

Clinical examinations were carried out under artificial light using plane surface dental mirrors and CPI probes. Dental caries was diagnosed according to the World Health Organization criteria (WHO, 2013). The erupting first permanent molars were recorded using the modified International Caries Detection Assessment System (ICDAS). The erupting stage of the first permanent molars was also recorded. To ensure the validity and reliability of the data collection, eight examiners were trained and calibrated. 5% of the subjects were selected randomly for repeated tests by examiners to measure their consistency. Before the oral examination, the children were instructed by dentists to brush their teeth at school. The examiners dried the surface of the teeth with cotton rolls and swabs, with the students in a supine position. No radiographic examination was performed. After the caries examination, a report was sent to the child’s caretakers to inform them if the child needed treatment.

### Questionnaire

The questionnaire was designed based on the one used in the third Chinese national oral health survey. The questionnaire was completed by each child’s parents or guardians, and included the following aspects: (a) child’s family background (gender, whether the child is the only one child in the family, and parental educational background); (b) oral health-related behavior (sweets intake, frequency of toothbrushing, fluoride toothpaste usage and dental visits); (c) oral health awareness and attitudes of parents or guardians. We scored the answers of oral health awareness and attitudes. If the answer was correct, one point was added; if wrong, one point was subtracted. If the parents or guardians were unsure of the answer, they were given zero points. The final scores were the arithmetic sum of all the points. A higher score indicated that parents or guardians had better oral health awareness or more active attitudes towards oral health care.

### Data analysis

A statistical software package (IBM SPSS Statistics, version 20) was used for data analysis. Caries prevalence (in %) and mean dmft score were calculated. An independent sample *t*-test (2 categories) and one-way ANOVA (more than 2 categories) were used to assess the statistical significance of the differences in the dental caries experience (mean dmft score). The Chi-square test was used to compare proportions. Binary logistic regression was performed to investigate the effects of the independent variables studied on the child’s dental caries experience. The dependent variable was dichotomized into dmft = 0 and dmft > 0. The independent variables were geographical region (Beizhen, Dahua, Linxia, Linxiang), gender, the only one child in the family (yes, no), father’s educational background (low, median, high), mother’s educational background (low, median, high), toothbrushing habits (≥2/d, <2/d), sweet consumption (≥2/d, 1/d, < 1/d), oral health awareness (good, median, poor), and attitudes (good, median, poor). Cohen’s Kappa statistics was used to evaluate the inter-examiners’ variability for dental caries examination. The statistical significance level for all tests was set at 0.05.

## Results

### Distribution of the studied children

The four selected county-level cities were Beizhen in Liaoning, Dahua in Guangxi, Linxia in Gansu and Linxiang in Hunan Province. Beizhen, Dahua, Linxia, and Linxiang comprised of 17, 16, 10 and 13 districts, respectively. Twenty-three public primary schools in 8 districts in Beizhen, nine schools in eight districts in Dahua, 10 schools in five districts in Linxia and six schools in six districts in Linxiang were invited to participate in the study. In total, 4,936 children, including 2,544 boys (51.5%) and 2,392 girls (48.5%) were included in the caries examination and questionnaire. The distribution of these subjects is presented in Table 1.

**Table 1:** The distribution of the studied children.

Province	Area	County-level city	Number of districts	Number of districts selected	Number of schools selected	Boys	Girls	Total
Liaoning	Northern China	Beizhen	17	8	23	712	706	1,418
Guangxi	Southern China	Dahua	16	8	9	466	454	920
Gansu	Western China	Linxia	10	5	10	522	600	1,122
Hunan	Central China	Linxiang	13	6	6	844	632	1,476

### Caries experience of the studied children

Compared with the reference examiner’s results, the eight examiners demonstrated very good reliability for caries diagnosis, with kappa-coefficient values of 0.88, 0.90, 0.83, 0.94, 0.81, 0.88, 0.84 and 0.85, respectively, for the WHO criteria; 0.82, 0.88, 0.84, 0.84, 0.83, 0.85, 0.81 and 0.80, respectively, for the modified ICDAS. The intra-examiner consistencies reached over 97.0% agreements for all eight examiners. The prevalence and severity of the dental caries in the studied children are summarized in Table 2 (primary dentition) and Table 3 (permanent dentition). An overwhelming majority (87.7%) of students were affected by caries, with the mean number of decayed, missing and filled teeth of 6.04 ± 4.24.

**Table 2:** Dental caries experience and prevalence of primary teeth of surveyed children with different districts and genders.

Group		n	dt	mt	ft	dmft (SD)	*p* Value (F)^*^	dmft > 0 (%)	*p* Value (*χ*^2^)^*^
Region	Beizhen	1,418	6.00	0.37	0.05	6.42 (3.58)	*<0.001*	92.9	<0.001
	Dahua	920	7.38	0.05	0.09	7.52 (4.90)	(105.25)	88.4	(84.33)
	Linxia	1,122	4.23	0.08	0.08	4.40 (3.64)		80.9	
	Linxiang	1,476	5.59	0.12	0.19	5.89 (4.31)		87.3	
Gender	Boys	2,544	5.66	0.17	0.10	5.93 (4.25)	*0.696*	86.5	*0.009*
	Girls	2,392	5.81	0.17	0.11	6.09 (4.18)	(0.152)	88.9	(6.81)
Total		4,936	5.73	0.17	0.11	6.01 (4.22)		87.7	

**Notes.**

* *p* value of comparison of dmft.

** *p* value of comparison of dmft > 0 (%).

**Table 3:** Non-cavitated and cavitated dental caries experience and prevalence of first permanent molars of surveyed children with different districts and genders.

Group			Non-cavitated caries		Cavitated caries					
		*n*	D_A_S (SD)	*p* value (F)^*^	DS (SD)	FS (SD)	DFS (SD)	p value(F)^**^	DFT > 0 (%)	*p value* (*χ*^2^)^***^
Region	Beizhen	1,418	0.24 (0.76)	<0.001	0.03 (0.25)	0.03 (0.30)	0.06 (0.39)	*0.02*	2.8	*0.02*
	Dahua	920	0.34 (0.99)	(40.73)	0.03 (0.28)	—	0.03 (0.28)	(3.16)	2.0	(10.11
	Linxia	1,122	0.07 (0.39)		0.02 (0.19)	—	0.02 (0.19)		1.0	
	Linxiang	1,476	0.09 (0.41)		0.03 (0.33)	—	0.03 (0.34)		2.0	
Gender	Boys	2,544	0.13 (0.56)	<0.001	0.03 (0.29)	0.01 (0.14)	0.03 (0.33)	*0.12*	1.7	*0.13*
	Girls	2,392	0.23 (0.76)	(102.89)	0.03 (0.25)	0.01 (0.18)	0.04 (0.30)	(2.40)	2.3	(2.35)
Total		4,936	0.18 (0.67)		0.03 (0.27)	0.01 (0.16)	0.04 (0.31)		2.0	

**Notes.**

* *p* value of comparison of D_*A*_S.

** *p* value of comparison of DFS.

*** *p* value of comparison of DFT > 0 (%).

In the primary dentition, the prevalence of caries was 87.7%, and the mean dmft score was 6.01 ± 4.22. The prevalence of caries was significantly different among different regions (*p* < 0.001). Beizhen and Dahua had higher caries prevalence. The prevalence of caries was higher in girls than in boys (*p* < 0.05).

Each child had an average of 5.13 erupted permanent teeth. In the permanent dentition, the prevalence of caries was 2.0% and the mean DFS score was 0.04 ± 0.31. All cavitated caries occurred in the first permanent molars. Carious tooth surfaces were identified as modified ICDAS code “A” to indicate initial caries and distinct visual change in enamel. The D_A_S score of non-cavitated caries in the first permanent molars reached 0.18 (SD, 0.67), which accounted for 1.47% of the erupted surfaces. The prevalence of both cavitated and non-cavitated caries was found to be significantly different among the different regions (*p* < 0.05). The prevalence of non-caries was higher in girls than in boys (*p* < 0.001).

### Results from the questionnaires

The response rate of questions was 98.4% to 99.8%. Caries experience (mean dmft score) according to the different contributing factors studied is shown in Table 4. Children who had siblings suffered more severe primary tooth decay than those who were the only one child in the family (*p* < 0.05). Caries experience also showed that higher educational background of the parents correlated with a lower mean dmft score of the primary teeth in their children (*p* < 0.001).

**Table 4:** *T*-test/ANOVA results with the dmft and DMFT scores as dependent variable respectively.

Group		*N*	Primary teeth		Permanent teeth	
			dmft (SD)	*p* value (F)^*^	DMFT (SD)	*p* value (F)^**^
Sibling	have	2,437	5.82 (4.11)	*0.005*	0.04 (0.29)	*<0.001*
	do not have	2,491	6.20 (4.32)	(7.915)	0.03 (0.22)	(14.768)
Father’s educational background	low (junior school or below)	2,347	6.23 (4.25)	*<0.001*	0.04 (0.28)	*0.17*
	median (senior school)	1,386	6.20 (4.17)	(761.893)	0.04 (0.29)	(0.236)
	high (college or above)	1,158	5.29 (4.13)		0.02 (0.17)	
Mother’s educational background	low (junior school or below)	2,676	6.27 (4.21)	*<0.001*	0.04 (0.29)	*0.073*
	median (senior school)	1,242	6.03 (4.26)	(904.825)	0.03 (0.24)	(0.352)
	high (college or above)	941	5.13 (4.04)		0.02 (0.17)	
Toothbrush habits	≥2/d	1,224	6.40 (4.52)	*<0.001*	0.02 (0.19)	*0.001*
	<2/d	3,710	5.88 (4.11)	(19.701)	0.04 (0.28)	(11.949)
Sweet consumption	≥2/d	590	6.80 (4.48)	*<0.001*	0.04 (0.29)	*0.345*
	1/d	1,541	6.16 (4.19)	(16.542)	0.04 (0.28)	(1.065)
	<1/d	2,784	5.75 (4.15)		0.03 (0.24)	
Oral health awareness	good (7∼8)	741	5.82 (4.21)	*0.248*	0.02 (0.19)	*0.363*
	median (4∼6)	2,481	6.10 (4.21)	(1.394)	0.03 (0.26)	(1.014)
	poor (≤3)	1,686	5.98 (4.23)		0.04 (0.28)	
Oral health attitude	good (3∼4)	4,489	6.00 (4.21)	*0.326*	0.03 (0.26)	*0.263*
	median (2)	330	5.97 (4.23)	(1.122)	0.05 (0.28)	(1.337)
	poor (≤1)	102	6.63 (4.49)		0.01 (0.10)	

**Notes.**

* *p* value of comparison of dmft.

** *p* value of comparison of DMFT.

Children who brushed their teeth at least twice daily had higher mean dmft score in primary dentition than those who brushed their teeth less than twice daily. According to the questionnaire, 95.6% of children used toothpaste in their daily brushing, of whom only 24.3% used fluoride toothpaste.

Overall, 43.4% of the studied children consumed sweets daily. The frequency of eating sweets was found to be positively associated with the mean dmft score in both primary and permanent dentitions. A statistically significant difference was found in primary dentition (*p* < 0.001).

Eight questions were used to assess the oral health awareness of the children’s parents or guardians and 4 questions were for their attitudes (Table 5). Only 7.5% of the responders answered all the questions correctly. “Pit and fissure sealant can prevent dental caries” and “Oral diseases can cause or exacerbate some systemic diseases” were unknown to most respondents, with the lowest correct rate of 27.1% and 35.2%, respectively. “Sugar can cause dental caries” was agreed by 86.9% of the responders, which was the highest correct rate. However, no statistically significant associations were found between the children’s caries experience and their parents’ oral health awareness and attitudes (*p* > 0.05).

**Table 5:** The percentage of response on questions of oral health knowledge and attitude (%).

	Agree	Disagree	Unknown
Oral health awareness			
1. Gum bleeding is normal when brushing your teeth.	12.1	77.7	10.2
2. Bacteria are one of the causes of inflammation of the gums.	79.0	6.5	14.5
3. Cleaning your teeth is not useful for preventing inflammation of the gums.	14.2	64.8	21.0
4. Dental caries are caused by bacteria on teeth.	74.9	7.1	18.0
5. Sweets can lead to tooth decay.	86.9	6.6	6.5
6. Fluoride is useless to dental protection.	8.1	47.2	44.7
7. Pit and fissure sealant can prevent dental caries of children	27.1	13.8	59.1
8. Oral diseases can cause or exacerbate certain systemic diseases.	35.2	10.3	54.5
Oral health attitudes			
1. Oral health is important to life	98.7	0.7	0.6
2. Regular oral examination is necessary.	92.6	2.2	5.2
3. Teeth are born good or bad, no correlation with the protection	4.8	93.3	1.9
4. We should rely mainly on ourselves to prevent oral diseases.	98.0	1.1	0.9

**Figure 1:**
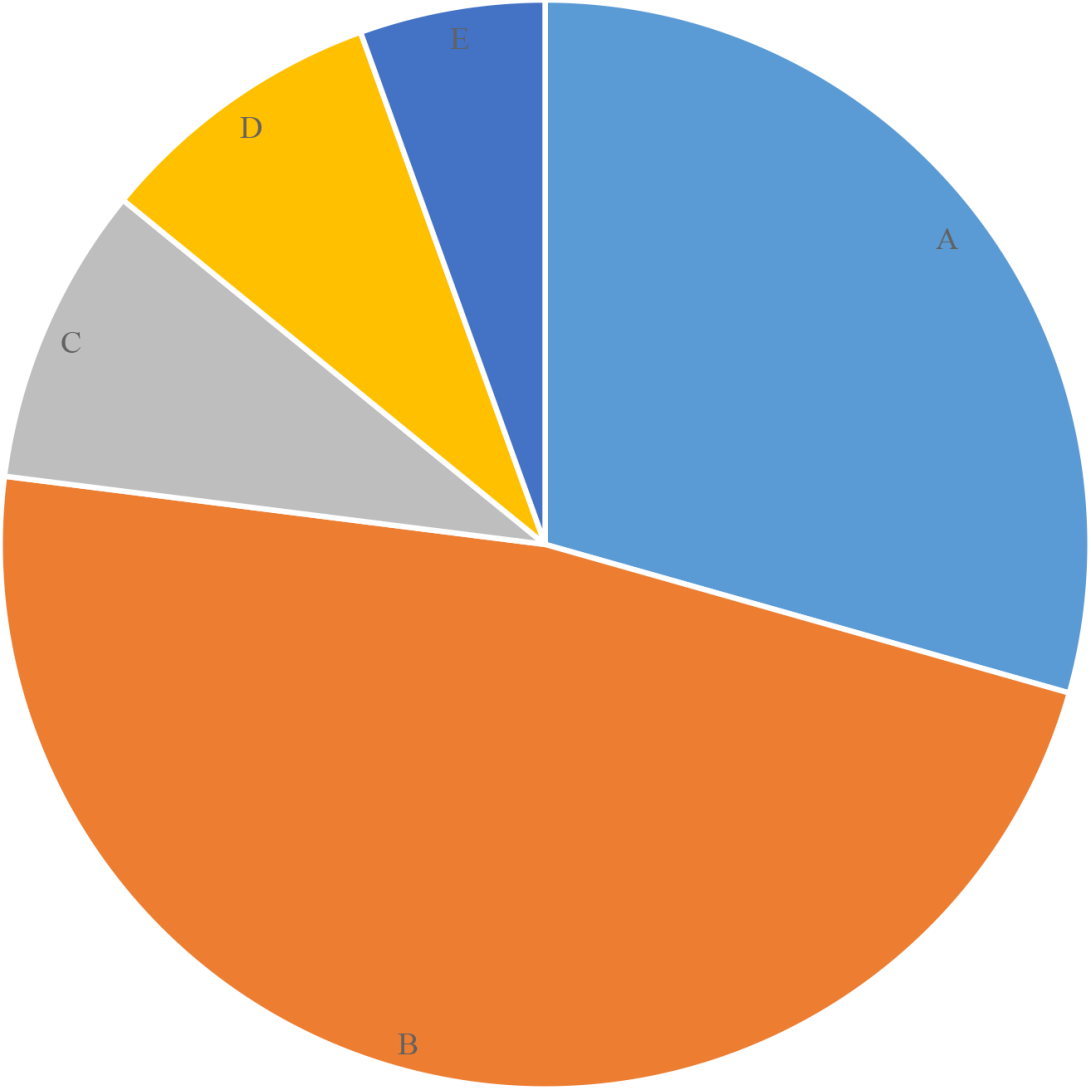
The percentage of reasons for dental visits among the surveyed children(%). (A) Consult and examination: 29.4. (B) Toothache or discomfort: 47.6. (C) Treatment: 8.9. (D) Regular examination for prevention: 8.6. (E) Others: 5.5.

Overall, 56.9% of the parents or guardians reported that their children suffered from toothache during the past 12 months, but only 27.6% of them visited the dentists. The main reason that children sought dental care was from oral pain or discomfort (48.6%). Only 9.0% of children received regular dental examinations and treatments for caries prevention (Fig. 1).

### Results from the binary logistic analysis

The results of the binary logistic analysis showed that regional and gender factors contributed to the caries experience of the studied children (*p* < 0.05). Compared with the caries experience of Linxiang, Beizhen was more severe and Linxia less severe. A higher caries experience was found in girls (Table 6).

## Discussion

Trends of dental caries in a population can only be observed from long-term epidemiological surveillance and surveys. In the past decades, some scholars have found that the prevalence of primary dental caries in 6-year-olds was approximately 74.37% to 76.7%, and the mean dmft score was approximately 3.71 to 3.94 (Yong Jiang, 1996; Zhang & Helderman, 2006; Jianzhong et al., 2012). The prevalence of permanent dental caries was approximately 2.98% (Jianzhong et al., 2012). In the present study, the prevalence of caries in primary teeth of 6-year-old children was significantly higher than the previous results, and the caries level appeared to be rising. Thus, in the past decade, the caries in primary teeth of 6-year-old children have rapidly increased. This is consistent with the results of the fourth Chinese national oral health survey in 2015 (Wang, 2018). This may be related to an increase consumption of sugar-rich foods. The consumption rate of sweet foods for children aged 6-11 increased from 23.1% in 2002 to 43.9% in 2012 (Liu, 2016).

**Table 6:** Binary Logistic regression analysis for the dental caries status of 6-year-old children.

Independent variable		B	S.E.	*p* value	Exp (B)	95% CI
District		1.937	0.222	*<0.001*	6.941	0.655–1.235
	Beizhen	0.796	0.143	*<0.001*	2.216	1.674–2.933
	Dahua	0.79	0.136	*0.561*	1.083	0.829–1.414
	Linxia	−0.429	0.119	*<0.001*	0.651	0.515–0.822
	Linxiang^a^					
Gender	Boys	−0.250	0.090	*0.006*	0.779	0.653–0.929
	Girls^a^					

**Notes.**

a Reference category.

Our study shows that regional and gender factors contribute to the caries experience of 6-year-old children. A higher prevalence of caries was found in Beizhen, Liaoning Province and Dahua, Guangxi Province, while the lowest was found in Linxia, Gansu Province. The trend in the distribution of caries according to regional difference is in consistency with the results of the third Chinese national oral health survey (Qi, 2008). This may be related to different diets and lifestyles in different regions. Previous investigations have found that children in Liaoning Province consume a large amount of sweets in various forms, and most children ate sweets before sleeping (Yin et al., 2008). Guangxi Province is a main sugar-producing area, and local residents have a habit of consuming sweets. Many school canteens sell a variety of candies, biscuits, carbonated drinks and other sugar-rich foods (Ming, Xiaojuan & Tang, 2015). The high prevalence of dental caries among children in Liaoning and Guangxi was associated with more sugar intake. In our study, children in Beizhen and Dahua were also found to consume large amounts of sweets with higher frequency. Almost all the studied children in Linxia were found to be of Hui ethnicity. Because of their Islamic religious beliefs, families and schools are strict with their diet and oral hygiene practices. They believe that purity is of the utmost importance in their life. Their daily sugar intake is low. They rinse their mouth with fresh water after eating and before reading the Quran to keep their oral cavity clean to show respect to God. As a result, people of Hui ethnicity tend to have better oral hygiene. This may explain the relatively low caries prevalence in Linxia. A previous study found that people in Hunan had unsatisfactory oral health awareness and used an improper toothbrushing technique (Zhengping Li, 2002). This may have led to the poor caries status in Linxiang.

There was no definitive conclusion about the relationship between gender and caries (Martinez-Mier & Zandona, 2013). The third and fourth Chinese national oral health surveys showed that boys had more caries than girls in primary dentitions of 5-year-olds, and the opposite was found in permanent dentitions of 12-year-olds (Qi, 2008; Wang, 2018). This finding was in agreement with the findings of most surveys (Liu, Zhou & Feng, 2016; Topaloglu-Ak & Eden, 2010). This study found that girls were more susceptible to caries than boys in both primary and permanent dentitions at the age of 6. This may be because girls’ permanent teeth erupt earlier than boys’, and the examined girls consumed more sweets than boys. As a result, the girls’ teeth were exposed to sweets earlier and had a greater chance of decaying.

In this study, the prevalence of permanent dental caries was 2.0% in 6-year-olds, while it reached 16.9% by the age of 7 (Quanchun et al., 2012), indicating that the incidence of caries increased very rapidly between the ages of 6 and 7. This finding was consistent with a previous study (Yaegaki et al., 1989). Our study showed that all permanent dental caries occurred on the first permanent molars in 6-year-olds, while the eruption rate of the first permanent molars was only 62.9%. Carvalho and Abernathy found that the first permanent molars were more susceptible to caries during the first 1–3 years after the eruption and the occlusal surfaces of the first permanent molars were particularly vulnerable to caries development at the age of 6 (Carvalho, Ekstrand & Thylstrup, 1989; Abernathy et al., 1986). At this time, an erupting permanent first molar lacks an antagonist contact. The complicated pits and fissures and the operculum covering the distal half of the first permanent molars allow for the accumulation and retention of dental plaque. In addition, the permanent molars are in the posterior region of the child’s mouth, which makes it further difficult for the child to properly clean this area. A study by Fontana & Zero showed that the caries experience was the best predictor of caries (Fontana & Zero, 2006). Other investigators found that the number of decayed primary molars may be correlated with caries increment in the permanent dentition in contrast to the number of decayed primary anterior teeth (Zhang & Helderman, 2006; Topaloglu-Ak & Eden, 2010). The prevalence of caries in primary teeth reported in this study was 87.7%, of which 72.0% occurred on primary molars. The severity of the caries of primary molars may also increase the risk of caries in the early erupting stage of the first permanent molars. However, this still needs to be further confirmed in a longitudinal study. All these factors may contribute to the initiation of the carious process before complete eruption has occurred.

Initial non-cavitated caries in the permanent dentition was suggested to be a predictor of caries in other studies (Seppä & Hausen, 1988; Steiner, Helfenstein & Marthaler, 1992). This study found that 1.47% of the erupted surfaces had already suffered initial non-cavitated caries in first permanent molars. Along with severe caries in primary molars and past caries experience, these studied children would be at great risk of dental caries in the future if preventive methods are not applied.

Sugar consumption by the children examined was high. However, it was not adequate to focus solely on sugar intake. The oral health behaviors and awareness of the studied children and their parents or guardians were also not satisfactory. In this study, the children who brushed teeth more than twice a day and who visited dentists often were those who had higher caries experience. It was plausible that children would not realize the importance of toothbrushing until they suffered dental caries, after which more rigorous dental care routines, such as brushing more than twice a day, were enforced in their households. Problem-oriented dental care behavior is common in China. This finding was also noted in previous studies (Jianzhong et al., 2012) (Liu, Zhou & Feng, 2016). Oral health awareness has significantly improved since the third Chinese national oral health survey (Qi, 2008), but there were still some misunderstandings and blind spots, mainly regarding the awareness of oral health prevention measures, including pit and fissure sealant and fluoride application. Our study was conducted in rural areas of China, where some parents migrate to cities for work and leave their child at home with grandparents. The elderly usually had poor oral health awareness and thus were unable to guide the child to form good oral health habits, which may have further aggravated the caries incidence of the child.

The time between eruption of the first permanent molars and the establishment of functional occlusion is a critical period to protect the permanent teeth from caries. Protection should begin as soon as the first permanent molar erupts rather than wait until the eruption is completed. Application of topical fluoride varnish and pit and fissure sealant should be popularized among children of appropriate age. Most children in China begin to go to primary school at the age of 6. This would be a desirable time for them to be instructed to establish correct oral health attitudes and behaviors at school. Effective professional protection and good oral health behavior will greatly reduce the risk of suffering permanent dental caries in the future. Moreover, caretakers’ awareness of oral health should be strengthened, which can help to promote the establishment and supervision of children’s oral health habits.

### Limitations of the study

In this study, we assessed the dental caries experience of the children from four county-level cities. There are several weaknesses in this study. First, the studied children were selected using the cluster sampling method, which may not be as representative as those selected through stratified sampling. Second, since radiographic examination was not performed, the initial proximal caries may not have been detected. Third, some questions were not answered by the parents or guardians, and statistical analysis was only performed for questions with answers. Although the excluded number was small, it may have affected statistical results. In addition, we were unable to ascertain the level of parental bias in responding to the self-reported questionnaires.

## Conclusions

The caries experience in primary dentition has significantly worsened. The first permanent molars were at high risk for dental caries. Regional and gender factors contributed to the caries experience of the examined children. It is critical to protect the permanent teeth from caries as soon as the first permanent molar erupts. A joint effort with the education department to promote oral health of the school-age children would be practicable and efficient. Meanwhile, the role of caretakers should also be emphasized.

##  Supplemental Information

10.7717/peerj.6997/supp-1 Supplemental Information 1 Clinical examination and questionnaire

## Additional Information and Declarations

### Data Availability

The following information was supplied regarding data availability: Raw data is available as a Supplemental File.
